# Willingness of Chinese Men Who Have Sex With Men to Use Smartphone-Based Electronic Readers for HIV Self-testing: Web-Based Cross-sectional Study

**DOI:** 10.2196/26480

**Published:** 2021-11-19

**Authors:** Gifty Marley, Gengfeng Fu, Ye Zhang, Jianjun Li, Joseph D Tucker, Weiming Tang, Rongbin Yu

**Affiliations:** 1 School of Public Health Nanjing Medical University Nanjing China; 2 The Social Entrepreneurship to Spur Health Project The University of North Carolina Project-China Guangzhou China; 3 Section of STD/AIDS Prevention and Control Jiangsu Provincial Center for Disease Control and Prevention Nanjing China; 4 Kirby Institute The University of New South Wales Sydney Australia; 5 Faculty of Infectious and Tropical Diseases London School of Hygiene and Tropical Medicine London United Kingdom; 6 Guangdong Second Provincial General Hospital Guangzhou China

**Keywords:** smartphone-based electronic reader, electronic readers, HIV self-testing, HIVST, self-testing, cellular phone–based readers, mHealth

## Abstract

**Background:**

The need for strategies to encourage user-initiated reporting of results after HIV self-testing (HIVST) persists. Smartphone-based electronic readers (SERs) have been shown capable of reading diagnostics results accurately in point-of-care diagnostics and could bridge the current gaps between HIVST and linkage to care.

**Objective:**

Our study aimed to assess the willingness of Chinese men who have sex with men (MSM) in the Jiangsu province to use an SER for HIVST through a web-based cross-sectional study.

**Methods:**

From February to April 2020, we conducted a convenience web-based survey among Chinese MSM by using a pretested structured questionnaire. Survey items were adapted from previous HIVST feasibility studies and modified as required. Prior to answering reader-related questions, participants watched a video showcasing a prototype SER. Statistical analysis included descriptive analysis, chi-squared test, and multivariable logistic regression. *P* values less than .05 were deemed statistically significant.

**Results:**

Of 692 participants, 369 (53.3%) were aged 26-40 years, 456 (65.9%) had ever self-tested for HIV, and 493 (71.2%) were willing to use an SER for HIVST. Approximately 98% (483/493) of the willing participants, 85.3% (459/538) of ever self-tested and never self-tested, and 40% (46/115) of unwilling participants reported that SERs would increase their HIVST frequency. Engaging in unprotected anal intercourse with regular partners compared to consistently using condoms (adjusted odds ratio [AOR] 3.04, 95% CI 1.19-7.74) increased the odds of willingness to use an SER for HIVST. Participants who had ever considered HIVST at home with a partner right before sex compared to those who had not (AOR 2.99, 95% CI 1.13-7.90) were also more willing to use an SER for HIVST. Playing receptive roles during anal intercourse compared to playing insertive roles (AOR 0.05, 95% CI 0.02-0.14) was associated with decreased odds of being willing to use an SER for HIVST. The majority of the participants (447/608, 73.5%) preferred to purchase readers from local Centers of Disease Control and Prevention offices and 51.2% (311/608) of the participants were willing to pay less than US $4.70 for a reader device.

**Conclusions:**

The majority of the Chinese MSM, especially those with high sexual risk behaviors, were willing to use an SER for HIVST. Many MSM were also willing to self-test more frequently for HIV with an SER. Further research is needed to ascertain the diagnostic and real-time data-capturing capacity of prototype SERs during HIVST.

## Introduction

Many countries, including China, have adopted HIV self-testing (HIVST) strategies to complement HIV testing services following the World Health Organization’s recommendation in 2016 [1]. Subsequently, various studies found the use of HIVST acceptable to key populations such as men who have sex with men (MSM) in China and around the world [2-5]. This strategy has also been shown to help reach hidden MSM who may have never accessed facility-based testing owing to fear of stigma and discrimination or other reasons [6,7]. Moreover, study participants have been cited to deem the privacy, convenience, and confidentiality associated with HIVST-facilitating factors for uptake, in addition to ease of use and short wait time [4,8].

HIVST continues to play a significant role in expanding HIV-testing services and was especially vital in retaining testing services during the COVID-19 outbreak when mobility was restricted [9-11]. For example, people in countries such as China, where HIVST has been approved for sale, could purchase kits online for doorstep delivery. This helped keep HIV testing services available and accessible during the pandemic period. However, upscaling HIVST to nationwide rollouts remains slow for many countries, including China, owing to existing gaps in distribution policies and integration strategies, persisting health worker concerns about low rates of user-initiated reporting of results, and lack of standardized reporting systems [12-15]. Although some studies found that the implementation of HIVST interventions where users can report their test results online (ie, texting the results to a health care worker on social media) was helpful to improve HIVST uptake and repeated HIV testing among Chinese MSM [16], reporting of posttest results remains largely dependent on provider-initiated routine follow-ups (such as home visits and reminder calling) [15,17-19]. Thus, the need for strategies that encourage user-initiated reporting of results at minimal/no extra cost to service providers requires further innovative measures.

Smartphone-based electronic readers (SERs) are smartphones adapted for health monitoring or clinical diagnostics and may include the use of external compatible hardware attachments (such as external optical lenses) to perform microscopic imaging [20,21]. SERs have become more popular in recent years because they consist of wearable heart rate monitors, temperature check apps, fitness products such as Fitbit watches, and blood glucose tracker apps [22,23]. Although the risks of private data leakage exist [24], the advantages SERs present for remote outpatient monitoring and early disease diagnosis cannot be understated [24,25]. For example, by using wearable sensors that feed into smartphone-based health apps, health care staff can monitor important physiological signs and activities of patients at home in real time from far-off facilities [26]. This could enable patients with chronic diseases to stay in care at home and is relatively cheaper compared to health facility admissions. Hence, SERs present an efficient cost-effective alternative to on-site clinical monitoring [27]. Furthermore, the emergence of SERs is vital to the expansion of decentralized infectious disease diagnostics [28], because unlike traditional laboratory diagnostics, they are portable and easy to use with minimal training required.

SERs are not a replacement for traditional laboratory diagnostics, but they could serve as preliminary diagnostic systems for early infectious disease diagnosis, especially in remote areas [20,21,29,30]. SERs such as smartphone-based bioimaging, smartphone-based bioanalyzers, and smartphone-based immune biosensors have been proven useful in point-of-care diagnostics [29,31,32]. Some pilot studies have also evaluated prototype SER use in point-of-care and found them to have comparable diagnostic capacity to traditional laboratory diagnostics. For example, a prototype smartphone-based rapid diagnostic test scanner was found capable of interpreting rapid diagnostic tests of 2 malaria species with comparable accuracy to standard visual interpretation [33,34]. Another study found a prototype SER capable of analyzing unprocessed liquid semen samples and providing users with a World Health Organization standard semen quality evaluation report with 98% accuracy [35]. Furthermore, other studies have shown prototype SERs capable of interpreting HIV rapid diagnostic test results with good accuracy and highlighted their capacity to capture real-time data [36,37]. Hence, coupled with recent advancements in smartphone computing, artificial intelligence, and open-source operating systems, SERs could be potential substitutes for bridging patient–health care provider barriers, and they could facilitate HIVST results reporting and active posttest counseling services through real-time data capturing.

However, as an emerging innovative tool without public implementation, it is not yet clear if HIVST users will be willing to use SERs for HIVST. Hence, there is a need to investigate public willingness to use SERs and to identify the determining factors associated with HIVST users’ willingness to use SERs prior to adopting it. This study aimed to assess the willingness of MSM in the Jiangsu province of China to use an SER for HIVST through a web-based cross-sectional study.

## Methods

### Study Design

HIV prevalence among MSM in the Jiangsu province in the eastern part of China increased from 6.6% in 2015 [38] to about 8% in 2019, according to the Jiangsu Provincial Centers for Disease Control and Prevention (CDC) surveillance data. Between February and April 2020, we conducted a web-based cross-sectional study among Chinese MSM in the Jiangsu province. The survey was conducted using a structured questionnaire hosted on a web-based survey platform (wjx.cn). Questions on sociodemographic data, sexual behaviors, and HIV testing experiences were adopted from a previous questionnaire used by our team in other MSM-related studies [39,40]. Questions on willingness to use smartphone-based readers were adopted from a previously used HIVST acceptability questionnaire and modified accordingly [41]. We pretested the questionnaire among 40 MSM purposively selected from a convenient sample of MSM clients who visited the Jiangsu Provincial CDC clinic for HIV testing services.

### Convenience Web-Based Sampling

To participate in the study, participants who clicked the survey link had to satisfy some prespecified eligibility criteria, which included male sex at birth, be aged 16 years and older, had ever engaged in sex with other men, lived in the Jiangsu province, and be willing to provide informed consent to join the study. Willing participants who met the eligibility criteria had to agree to voluntarily participate in our study and for their provided data to be used strictly for research purposes. Participants who consented to this were redirected to the survey page to complete the questionnaire. The psychological state of the participants who met the eligibility criteria and provided informed consent was however not ascertained.

### Data Collection

This study solicited information on participants’ demographic variables, including their age, education level, marital status, household residency status, monthly income, and sexual orientation. Data solicited on sexual behaviors included sexual orientation disclosure to others, including sex workers, apart from sexual partners, number of sexual partners in the preceding 6 months, and usual places of meeting sexual partners (eg, pubs, night clubs, public bathrooms, online, through friends, others). Participants were also asked how often they used condoms during anal sex with their sexual partners in the preceding 6 months, if they ever participated in group sex/orgies (defined as sex with at least 2 men at the same time), and if they knew the HIV status of their sexual partners. We also collected information on the number of casual and regular male partner(s) in the preceding 6 months as well as the roles participants played during anal sex with regular and casual partners. HIV testing history was assessed with the questions: how often do you test for HIV, how many times did you test for HIV in the past year, how many times have you tested for HIV this year, do you know your HIV status, do you know the HIV status of your regular partner, and have you ever disclosed your HIV status to any of your sexual partners. Participants were also asked if they had ever self-tested for HIV, where they obtained HIVST kits, if they used HIVST for their first HIV testing, and if they had ever considered testing for HIV at home with a partner right before sex. Information on self-reported concerns about HIVST as well as willingness to report HIVST results by sending pictures of the used test kits to health care workers or researchers were also collected.

### Assessment of Willingness

Participants watched a short video showcasing a prototype SER prior to answering questions on willingness (Multimedia Appendix 1), in addition to a short introductory paragraph on SERs to provide context. The prototype consisted of a portable 3D printed case, which housed a light-emitting diode light source, a mini scanner, a Bluetooth tag, and a closing cap to ensure stability and safe hold during use. The detailed description of the prototype SER will be made available upon completion.

### SER Use

Self-testers need to connect the reader to a smartphone device via Bluetooth after turning on the reader. After installing the needed software and entering some basic biodata, users can proceed to uncap the reader, place the used HIVST kit into the reader slot following the direction (to know which part to insert), and close the cap to ensure the kit is secured in place. The reader would then scan the result display area of the test kit by using the embedded light-emitting diode scanner and highlight the display of the test lines (red strips) by reducing the background color value. This would enable the reader software to check whether the control line (C) and the test line (T) are displayed or not. The software will interpret the results as negative (C + T -), positive (C + T +), or invalid (C-T + / -) depending on which test lines are displayed. The reader software then transmits the results to a centralized system, which in turn feeds back the results to the smartphone and the CDC surveillance system concurrently. Participants were asked if they would be willing to use an SER for HIVST if it was provided for free. Subsequent questions asked if participants would recommend SERs to a sexual partner and whether having an SER would influence their HIVST frequency. Additional questions assessed participant opinions on factors that would facilitate and discourage them from opting to use SERs in future as well as how much they would be willing to pay to purchase such a reader device.

### Statistical Analysis

A descriptive analysis was conducted to summarize the sociodemographic characteristics, sexual behaviors, and HIV testing experience. Chi-squared test was used to test for statistically significant factors associated with MSM willingness to use smartphone-based HIVST readers if offered during self-testing. Individual correlation models were used to assess variables’ association with willingness to use an SER for HIVST. Factors that had a significant correlation with the dependent variable were entered into a multivariable logistic regression. Age, income, marital status, and level of education were considered potential confounders and adjusted in the model [8,42-44]. Statistical significance was defined at *P* values <.05, and all analyses were conducted using SPSS statistics version 23.0.0 (IBM Corp).

### Ethical Approval

Ethical approval was obtained from the ethics review committees at the Jiangsu Provincial CDC (project JSJK2019-B016-03). All participants provided virtual informed consent prior to participation in the study.

## Results

### Sociodemographic Characteristics and HIV Testing History

A total of 692 completed surveys were included in the data analysis. The majority of the participants (369/692, 53.3%) were aged between 26 and 40 years (median age 31-40 years), 30.5% (211/692) were older than 40 years, and 16.2% (112/692) were 25 years old and younger. Of all the participants, 67.5% (467/692) self-identified as homosexuals, 51.3% (355/692) had a college or university degree, and 50.9% (352/692) earned more than US $780 monthly. Most participants had disclosed their sexual orientation to others apart from their sexual partners (552/692, 79.8%), and the majority met sexual partners through web-based platforms (594/692, 85.8%). Many participants (437/692, 63.2%) had at least one regular sexual partner and 26% (180/692) reported having multiple temporary sexual partners in the preceding 6 months. Unprotected anal intercourse was more frequent with regular than with causal partners in the last 6 months (184/692, 26.6% vs 75/692,10.8%, respectively) (Table 1).

**Table 1:** Descriptive analysis of the sociodemographic characteristics and sexual history of men who have sex with men in China in 2020 (N=692).

Variables			Values, n (%)
**Age (years)**			
	≤25	112 (16.2)	
	26-40	369 (53.3)	
	>40	211 (30.5)	
**Marital status**			
	Single	340 (49.1)	
	Married	239 (34.5)	
	Divorced/separated	113 (16.3)	
**Highest education level**			
	High school	295 (42.6)	
	College/University	355 (51.3)	
	Postgraduate	42 (6.1)	
**Monthly income (USD)**			
	<230	28 (4)	
	230-780	312 (45.1)	
	>780	352 (50.9)	
**Sexual identity**			
	Homosexual	467 (67.5)	
	Bisexual	211 (30.5)	
	Unsure	14 (2)	
**Disclosed sexual orientation to others apart from sexual partners**			
	Yes	552 (79.8)	
	No	140 (20.2)	
**Places you usually meet sexual partners**			
	Hotspots (ie, pubs, bath houses, massage parlor, etc)	142 (20.5)	
	Public places (eg, parks, public restroom)	56 (8.1)	
	Online (ie, dating websites, social media)	594 (85.8)	
	Met through family/friends	180 (26)	
	Don’t search sexual partners	87 (12.6)	
**Unprotected anal intercourse in the last 6 months**			
	Yes	202 (29.2)	
	No	392 (56.6)	
	Have had no sexual partners	98 (14.2)	
**Number of regular sexual partners in the last 6 months**			
	None	255 (36.8)	
	1	348 (50.3)	
	≥2	89 (12.9)	
**Role during sex with regular partner**			
	Insertive (n=437)	232 (53.1)	
	Both (n=437)	131 (30)	
	Receptive (n=437)	74 (16.9)	
	Had no regular sexual partners (N=692)	255 (36.8)	
**Unprotected anal intercourse** **with regular partners**			
	Yes (n=437)	184 (42.1)	
	No (n=437)	253 (57.9)	
	Had no regular sexual partners (N=692)	255 (36.8)	
**Number of temporary partners in the last 6 months**			
	None	378 (54.6)	
	1	134 (19.4)	
	≥2	180 (26)	
**Role during sex with temporary partner**			
	Insertive (n=314)	111 (35.4)	
	Both (n=314)	69 (22)	
	Receptive (n=314)	84 (26.8)	
	Had no casual partners (N=692)	378 (54.6)	
	Refused to answer (n=314)	50 (15.9)	
**Unprotected anal intercourse** **with casual partner**			
	Yes (n=314)	75 (28.4)	
	No (n=314)	189 (71.6)	
	Had no casual partners (N=692)	378 (54.6)	
	Refused to answer (n=314)	50 (15.9)	

Most participants (495/692, 71.5%) had tested for HIV more than twice in the past year; 65.9% (456/692) had ever used an HIV self-test kit, of which 42.5% (194/456) used it for their first HIV test. The majority of the participants (560/692, 80.9%) had also considered home testing with their partner right before sex. Table 2 shows further descriptive details of participants’ HIV testing history.

**Table 2:** Descriptive analysis of participants’ HIV testing habits and self-testing experience in China in 2020.

Variables		Values, n (%)
**How often do you test for HIV? (N=692)**		
	3 months	398 (57.5)
	6 months	140 (20.2)
	1 year or more	154 (22.3)
**Number of times tested for HIV last year (N=692)**		
	Never	80 (11.6)
	Once	117 (16.9)
	≥Twice	495 (71.5)
**Know your HIV status (N=692)**		
	Yes	642 (92.8)
	No	50 (7.2)
**Ever self-tested for HIV (N=692)**		
	Yes	456 (65.9)
	No	156 (22.5)
	Never heard of self-testing	80 (11.6)
**Used HIV self-testing kit for first HIV test**		
	Yes (n=456)	194 (42.5)
	No (n=456)	262 (57.5)
**Ever considered testing for HIV at home prior to a sexual encounter (N=692)**		
	Yes	560 (80.9)
	No	132 (19.1)
**Concerns when you use HIV self-testing (N=692)**		
	Is it working correctly?	237 (34.2)
	Time to wait for results to appear	204 (29.5)
	Results accuracy	201 (29)
	Read the results correctly	276 (39.9)
**Comfortable sharing a picture of HIVST results with health worker for reporting (N=692)**		
	Yes	347 (50.1)
	No	345 (49.9)

### Factors Associated With Willingness to Use an SER

Of the 692 participants, 493 (71.2%) were willing to use an SER during HIVST, 115 (16.6%) were unwilling, and 84 (12.1%) were unsure of their willingness. The majority of the willing participants (428/493, 86.8%) and few of unwilling participants (45/115, 39.1%) agreed they would recommend SERs to their sexual partners for HIVST. Additionally, 98% (483/493) of the participants agreed that having an SER would increase their HIVST frequency. Willing participants cited the following factors as the major facilitators that would encourage their use of an SER: obtaining accurate self-test results (328/493, 66.5%), ease of use (300/493, 60.9%), and short wait time of 15-20 minutes for results (251/493, 50.9%). Alternatively, cost of the reader (259/493, 52.5%) and fear of test results leaking to others (214/493, 43.4%) were deemed barriers. Most willing participants preferred to purchase SERs from local offices of the CDC (375/493, 76.1%), pharmacies/supermarkets (200/493, 40.6%), and vending machines (192/493, 38.9%). The acceptable cost for an SER was less than US $4.30 (239/493, 48.5%), although 32% (158/493) were willing to pay US $8.70 or more. Among the 18.9% (115/692) unwilling participants, 61.7% (71/115) would consider using SERs owing to its ease of use and less wait time of 15-20 minutes for results (57/115, 49.6%), while having never heard of the reader (59/115, 51.3%) and purchase cost (43/115, 37.4%) would deter use. Likewise, many unwilling participants preferred to buy SERs from local CDC offices (72/115, 62.6%) and hospitals/clinics (60/115, 52.2%) at an acceptable cost of less than US $4.30 (72/115, 62.6%) (Table 3).

**Table 3:** Factors associated with willingness to use smartphone-based electronic readers among Chinese men who have sex with men.

Variables		Willing to use reader			Total (n=608), n (%)		*P* value	
		Yes (n=493), n (%)	No (n=115), n (%)					
				
**Would recommend HIV self-testing kit reader to sexual partner**								<.001^a^
	Yes	428 (86.8)	45 (39.1)	473 (77.8)				
	No	65 (13.2)	70 (60.9)	135 (22.2)				
**HIV self-testing kit reader would increase your HIV testing frequency**								<.001^a^
	Yes	483 (98)	46 (40)	529 (87)				
	No	10 (2)	69 (60)	79 (13)				
**Concerned about reading HIV self-testing results correctly**								.001^b^
	Yes	174 (35.3)	60 (52.2)	234 (38.5)				
	No	319 (64.7)	55 (47.8)	374 (61.5)				
**Facilitators of smartphone-based electronic reader use**								
	Easy to use	300 (60.9)	71 (61.7)	371 (61)		.86		
	You don't need to interpret the results of HIV self-testing yourself	213 (43.2)	11 (9.6)	224 (40.1)		<.001^a^		
	15-20 minutes to find out the results of HIV self-testing	251 (50.9)	57 (49.6)	289 (47.5)		.79		
	Get accurate results	328 (66.5)	46 (40)	374 (61.5)		<.001^a^		
	Referral services can be implemented online if the test results are positive	200 (40.6)	49 (42.6)	249 (41)		.69		
**Barriers to smartphone-based electronic reader use**								
	Never heard of it	176 (35.7)	59 (51.3)	235 (38.7)		.002^b^		
	Worried about accuracy	141 (28.6)	25 (21.7)	166 (27.3)		.14		
	Cost	259 (52.5)	43 (37.4)	302 (49.7)		.003^b^		
	Worried about test results leak to others (including to health care workers)	214 (43.4)	42 (36.5)	256 (42.1)		<.001^a^		
	Worried that others will see the test results on the screen or on their phone	178 (36.1)	31 (27)	209 (34.4)		.002^b^		
	Worried about personal data being misused	187 (37.9)	26 (22.6)	213 (35)		<.001^a^		
	Waiting anxiety and fear of positive results	105 (21.3)	N/A^c^	105 (17.3)				
**Where will you like to obtain a self-test reader**								
	Pharmacy, supermarket, etc	200 (40.6)	11 (9.6)	211 (34.7)		<.001^a^		
	Web-based mall	167 (33.9)	54 (47)	221 (36.3)		.009^b^		
	Hospital or clinic	153 (31)	60 (52.2)	213 (35)		<.001^a^		
	Vending machines	192 (38.9)	45 (39.1)	237 (39)		.97		
	Local Centers for Disease Control and Prevention office	375 (76.1)	72 (62.6)	447 (73.5)		.003		
**How much are you willing to pay for a HIV self-testing kit reader (USD)**								.003^b^
	<4.60	239 (48.5)	72 (62.6)	311 (51.2)				
	4.70-9.40	96 (19.5)	16 (13.9)	112 (18.4)				
	>9.50	158 (32)	27 (23.5)	185 (30.4)				

^a^Statistically significant at *P*<.01

^b^Statistically significant at *P*<.05.

^c^N/A: not applicable.

Furthermore, 78.3% (299/382) of the participants who had “ever self-tested” for HIV and 79.3% (124/156) “never self-tested” participants were willing to use SERs. The majority of the “ever self-tested” and “never self-tested” participants (311/382, 81.4% and 124/156, 79.5%, respectively) would recommend the SERs to their partners and agreed that having an SER would increase their HIVST frequency (335/382, 87.7% and 124/156, 79.5%, respectively). Willing “ever self-tested participants” deemed SERs’ wait time of 15-20 minutes (263/382, 68.8%), ease of use (249/382, 65.2%), and result accuracy (233/382, 61%) to be facilitating factors. Cost of reader (214/382, 56%), worry about test results leaking to others (204/382, 53.4%), and concerns about personnel data being misused (148/382, 38.7%) were deemed barriers.

Among the “never self-tested” participants, however, obtaining accurate results (92/156, 59%) and ease of use (84/156, 53.8%) were key facilitators, while having never heard of SERs (83/156, 53.2%), worry about test results leaking to others (54/156, 34.6%), and concerns about personnel data being misused (54/156, 34.6%) were deemed barriers. The majority of both “self-tested” and “never self-tested” participants preferred to buy SERs from their local CDC offices (293/382, 76.7% and 124/156, 79.5%, respectively), and 48.3% (260/538) were willing to pay less than 30 RMB (approximately US $4.60) for a reader (Table 4).

**Table 4:** Factors associated with the willingness of ever self-tested and never self-tested Chinese men who have sex with men to use smartphone-based electronic readers for HIV self-testing.

Variables			Self-tested (n=382), n (%)	Never self-tested (n =156), n (%)	Total (n=538), n (%)		*P* value		
**Would recommend HIV self-testing kit reader to sexual partner**								.63	
	Yes	311 (81.4)		124 (79.5)	435 (80.9)				
	No	60 (15.7)		32 (20.5)	92 (17.1)				
	Unsure	11 (2.9)		N/A^a^	11 (2)				
**HIV self-testing kit reader would increase your HIV testing frequency**								.02^b^	
	Yes	335 (87.7)		124 (79.5)	459 (85.3)				
	No	11 (2.9)		32 (20.5)	43 (8)				
	Unsure	36 (9.4)		N/A	36 (6.7)				
**Facilitators of HIV self-testing reader use**									
	Easy to use	249 (65.2)		84 (53.8)	333 (61.9)		.01^b^		
	You don't need to interpret the results of HIV self-testing yourself	155 (40.6)		52 (33.3)	207 (38.5)		.12		
	15-20 minutes to find out the results of HIV self-testing	263 (68.8)		28 (17.9)	291 (54.1)		.001^c^		
	Get accurate results	233 (61)		92 (59)	325 (60.4)		.66		
	Referral services can be implemented online if the test is reactive	173 (45.3)		46 (29.5)	219 (40.7)		.001^b^		
**Barriers to HIV self-testing reader use**								<.001^c^	
	Never heard of it	135 (35.3)		83 (53.2)	218 (40.5)				
	Worried about accuracy	127 (33.2)		26 (16.7)	153 (28.4)				
	Cost of device	214 (56)		54 (34.6)	268 (49.8)				
	Worry about test results leaking to others (including to health workers)	204 (53.4)		54 (34.6)	258 (48)				
	Worry about personal data being misused	148 (38.7)		33 (21.2)	181 (33.6)				
	Waiting anxiety and fear of positive results	105 (27.5)		N/A	105 (19.5)				
**Where will you like to obtain a self-test reader?**									
	Shops (pharmacy, supermarket, mall, etc)	99 (25.9)		61 (39.1)	160 (29.7)		.04^b^		
	Web-based mall	139 (36.4)		33 (21.2)	172 (32)		.001^b^		
	Hospital or clinic	117 (30.6)		79 (50.6)	196 (36.4)		<.001^c^		
	Vending machines	124 (32.5)		77 (49.4)	201 (37.4)		<.001^c^		
	Local Centers for Disease Control and Prevention office	293 (76.7)		124 (79.5)	417 (77.5)		.57		
**How much are you willing to pay for a HIV self-testing kit reader? (USD)**								<.001^c^	
	<4.60	174 (45.5)		86 (55.1)	260 (48.3)				
	4.70-9.40	58 (15.2)		54 (34.6)	112 (20.8)				
	≥9.50	150 (39.3)		16 (10.3)	166 (30.9)				

^a^N/A: not applicable.

^b^Correlation significant at *P*<.05.

^c^Correlation significant at *P*<.01.

### Predictors of MSM Willingness to Use an SER

MSM participants who engaged in unprotected anal intercourse with regular partners compared to those who consistently used condoms (adjusted odds ratio [AOR] 3.04, 95% CI 1.19-7.74) were more likely to be willing to use SERs for HIVST. Participants who had ever considered self-testing for HIV at home with a partner right before sex compared to those who had not (AOR 2.99, 95% CI 1.13-7.90) were also more willing to use an SER for HIVST. However, participants who played receptive roles during anal intercourse compared to those who played insertive roles (AOR 0.05, 95% CI 0.02-0.14) were less likely to be willing to use an SER for HIVST. Although not statistically significant, participants older than 40 years compared to participants 25 years old and younger (AOR 1.57, 95% CI 0.50-4.94), participants with a postgraduate level of education compared to participants who completed only high school (AOR 1.22, 95% CI 0.31-4.73), and participants who tested for HIV every 3 months compared to those who tested once a while (AOR 2.44, 95% CI 0.95-6.31) were also more willing to use an SER for HIVST. Table 5 further summarizes the details on predicting the factors associated with the willingness of MSM to use SERs.

**Table 5:** Logistic regression analysis of the factors predicting willingness to use smartphone-based electronic readers.

Variables				Total (n=608), n (%)		Willing to use readers					Adjusted odds ratio^a^ (95% CI)			*P* value
						Yes (n=493), n (%)		No (n=115), n (%)						
					
**Demographics characteristics**														
	**Age (years)**													
		26-40	330 (54.3)		258 (52.3)		72 (62.6)		0.61 (0.24-1.52)			.29		
		>40	187 (30.8)		163 (33.1)		24 (20.9)		1.57 (0.50-4.94)			.44		
		≤25	91 (15)		72 (14.6)		19 (16.5)		1					
	**Marital status**													
		Single	296 (48.7)		234 (47.5)		62 (53.9)		1.81 (0.68-4.76)			.23		
		Married	215 (35.4)		181 (36.7)		34 (29.6)		2.13 (0.81-5.63)			.13		
		Divorced/separated	97 (16)		78 (15.8)		19 (16.5)		1					
	**Highest education level**													
		College/University	314 (51.6)		256 (51.9)		58 (50.4)		0.69 (0.32-1.45)			.32		
		Postgraduate	40 (6.6)		32 (6.5)		8 (7)		1.22 (0.31-4.73)			.78		
		High school	254 (41.8)		205 (41.6)		49 (42.6)		1					
	**Monthly income (USD)**													
		<230	24 (3.9)		17 (3.4)		7 (6.1)		2.23 (0.32-15.48)			.42		
		230-780	273 (44.9)		225 (45.6)		48 (41.7)		1.06 (0.53-2.10)			.87		
		>780	311 (51.2)		251 (50.1)		60 (52.2)		1					
**Sexual behaviors**														
	**Role during sex with regular partner in the last 6 months**													
		Both	168 (27.6)		120 (24.3)		48 (41.7)		0.99 (0.39-2.51)			.98		
		Receptive	101 (16.6)		41 (8.3)		60 (52.2)		0.05 (0.02-0.14)			<.001^b^		
		Insertive	185 (30.4)		169 (34.3)		16 (13.9)		1					
	**Unprotected anal intercourse with regular partner** **in the last 6 months**													
		Yes	173 (28.5)		155 (31.4)		18 (15.7)		3.04 (1.19-7.74)			.02^c^		
		No	364 (59.9)		278 (56.4)		86 (74.8)		1					
	**Ever considered self-testing at home prior to sex**													
		Yes	476 (78.3)		402 (81.5)		74 (64.3)		2.99 (1.13-7.90)			.03^c^		
		No	132 (21.7)		91 (18.5)		41 (35.7)		1					
**HIV testing history**														
	**How often do you test for HIV?**													
		3 months	338 (55.6)		285 (57.8)		53 (46.1)		2.44 (0.95-6.31)			.07		
		6 months	130 (21.4)		98 (19.9)		32 (27.8)		0.47 (0.17-1.33)			.16		
		1 year or more	140 (23)		110 (22.3)		30 (26.1)		1					

^a^Adjusted for age, income, marital status, and level of education.

^b^Significant at *P*<.01.

^c^Statistically significant at *P*<.05.

## Discussion

### Principal Results

User-initiated reporting of results and linkage to care after self-testing are persistent barriers to HIVST uptake and scale-up. This study extends existing literature on the use of SERs as it explores MSM opinions about SERs and their willingness to use it for HIVST. Our findings showed that the majority of the Chinese MSM were willing to use SERs for HIVST. In addition, MSM with high sexual risk behaviors and those who had ever considered self-testing were more willing to use an SER for HIVST. Furthermore, SERs could help increase HIVST uptake and increase testing frequency among both ever self-tested and never self-tested MSM. Finally, most MSM were willing to pay less than US $4.30 for an SER and preferred to obtain it from their local CDC office.

We found that most participants were willing to use an SER for HIVST. Our findings showed that the majority of MSM (493/608, 81.1%), including 78.3% (299/382) of ever self-tested MSM and 79.5% (124/156) of never self-tested participants, were willing to use an SER for HIVST. Our finding concurs with findings of a recent study that found the use of a prototype SER for HIVST acceptable to MSM and transgender women participants [45]. The study attributed the high acceptability to the capacity of the prototype SER to save as well as share HIVST results with partners and health care providers [45]. We, however, speculate that the observed high willingness in our study may be due to HIVST users’ need to correctly read test results with assured accuracy. This explanation is plausible as 39.9% (276/692) of the participants in our study reported having concerns about their ability to accurately read and understand self-test results. Although this is a preliminary study, our findings provide some foundational evidence to trigger further research on the role of SERs in bridging HIVST-related gaps and expanding HIVST uptake. There is also a need for future studies to evaluate the real-time data-capturing capacity of prototype SERs and assess their functional integration into existing health systems.

In addition, MSM with higher sexual risk behaviors were more willing to use an SER for HIVST. Compared to participants who consistently used condoms, MSM who engaged in unprotected anal intercourse were more willing to use an SER for HIVST. This could be because MSM with higher sexual risk behaviors test for HIV more frequently owing to a perceived sense of being at higher risk for HIV infection [17,46]. Further, in support of this explanation, previous studies have found MSM who report inconsistent condom use with sexual partners to be more likely to have tested for HIV recently and opt for self-testing [4,47]. We also speculate that frequent testers are more likely to opt for HIVST for convenience and privacy. Therefore, having an SER to accurately interpret results will be deemed an added advantage. We also found that MSM who had ever considered self-testing at home with a partner right before sexual intercourse were willing to use an SER for HIVST. Therefore, it is possible that having a perceived sense of high risk to HIV and wanting to use HIVST plays a role in determining MSM willingness to use SERs. Further research is needed to better understand the dynamics of factors that predict willingness to use SERs among MSM and other HIVST users. We also recommend further research into behavioral factors that predict willingness to use SERs among different populations to inform the promotion of SERs in future.

Our findings showed that SERs could improve HIVST uptake among MSM. The majority of the “ever self-tested” and “never self-tested” participants in our study reported that having a reader would increase their HIVST frequency. However, less than half of the “unwilling” participants (46/115, 40%) reported the same. Nonetheless, this is still an important observation as our findings showed a current HIVST uptake rate of 65.8% (456/692) among the participants. This rate is still below the optimal coverage although it is an increase from the observed 37.2% in 2014 [48]. In addition, although many studies have proven users capable of properly undertaking HIVST [4,6,8], user concerns about their capacity to accurately read and interpret results still persist [15,49]. Therefore, this technology presents an opportunity to expand HIV testing among MSM and should be further investigated. For the majority of the unwilling participants (59/115, 51.3%) and never self-tested participants (83/156, 53.2%), never having heard of SERs could be a major barrier to use. Therefore, we recommend that public education on SER be undertaken and information about SERs be made readily available and accessible prior to SER introduction. Policies that standardize emerging HIVST reporting systems to ensure their smooth integration into existing health reporting structures and guard against personal data abuse are also needed.

The cost and place of purchase are vital to the promotion of SERs. Findings from our study showed that 51.2% (311/608) of MSM were unwilling to pay more than US $4.60 for an SER. Similarly, the majority of both never self-tested participants (86/156, 55.1%) and unwilling participants (72/115, 62.6%) were also unwilling to pay more than US $4.30 for an SER. This result is similar to the findings of previous studies that have shown purchase cost to be a deterring barrier to HIVST uptake [6,50]. The majority of the participants also preferred purchasing SERs from local CDC offices. This concurs with observations from previous HIVST acceptability studies in China [41,51]. We could attribute this to the notion that Chinese MSM trust the CDC owing to their involvement in the delivery of key population–centered HIV interventions [52,53]. It is also possible that the recruiting community-based organizations’ existing partnership with the CDC may have contributed to this preference. Therefore, in addition to local CDC offices, SERs should be made available at clinics, pharmacies, and web-based shops to facilitate accessibility to hidden populations when they are adopted. Furthermore, government agencies could team up with device manufacturers to minimize manufacturing cost and subsidize purchase cost to encourage uptake. Further, smartphone manufacturers should consider incorporating rapid tests reading functions and apps into emerging phone gadgets to minimize, if not eliminate, the extra cost of obtaining external SERs.

Our study has many implications as it evaluates the willingness of self-testers to use an emerging tool of importance to HIVST. Our findings highlight lay users’ expectations of SERs in HIVST that could inform manufacturers of specifications that will enhance SERs for better integration into HIVST programs. For research, future randomized controlled studies should assess the feasibility and acceptability of SERs among other population subtypes by using larger sample sizes. Furthermore, studies that seek to evaluate the sensitivity and specificity of SERs during HIVST by users should be conducted. Our study findings also support recommendations of previous studies that SERs should be embedded with cloud-based data functions. We also showcase the need for further evaluation of SER uses in real-time data capturing and monitoring. Lastly, quality assurance policies and guidelines are needed to inform the incorporation of SERs into HIVST programming as well as to standardize the manufacture and distribution of SERs.

### Limitations

Our study has some limitations. First, as this was a web-based cross-sectional study, the MSM sample size may be unrepresentative of the larger Jiangsu MSM population. Further, owing to the convenience sampling method employed, our study findings may be ungeneralizable. However, our findings still serve as preliminary data to further guide future research on the acceptability of SERs for HIVST. Second, as participants had limited familiarity with SERs and the query assessed willingness on the preconditioned clause that SER will be provided for free, the results on the willingness to use it and secondary distribution may have been biased. Nonetheless, our findings showed that MSM were willing to purchase SERs and highlighted important features required to encourage SER use in HIVST programming. Third, our investigation addressed sensitive questions that may have led to the misreporting of personal sexual risk and HIV testing behavior data owing to social desirability bias. Lastly, the mental status and capacity of eligible participants who provided informed consent were not assessed prior to administering the survey. Therefore, we recommend further studies to be conducted to explore the acceptance of the HIVST reader results among other subpopulation types prior to public implementation.

### Conclusion

Regardless of the limited knowledge, many Chinese MSM, especially those with high sexual risk behaviors, are willing to use SERs for HIVST. In addition, the majority of both ever self-tested and never self-tested MSM are willing to self-test more frequently with SERs. Therefore, SERs could facilitate HIVST uptake and scale-up among MSM. However, appropriate pricing and safe and anonymous procurement venues are key to facilitating SER uptake among key populations. Further research is needed to validate the uptake of SERs for HIVST among other key population subtypes.

## Appendix

Multimedia Appendix 1 Prototype smartphone-based electronic reader in use.

## Abbreviations

AOR: adjusted odds ratio
CDC: Centers for Disease Control and Prevention
HIVST: HIV self-testing
MSM: men who have sex with men
SER: smartphone-based electronic reader
